# Functional Characterization of a *Dendrobium officinale* Geraniol Synthase DoGES1 Involved in Floral Scent Formation

**DOI:** 10.3390/ijms21197005

**Published:** 2020-09-23

**Authors:** Conghui Zhao, Zhenming Yu, Jaime A. Teixeira da Silva, Chunmei He, Haobin Wang, Can Si, Mingze Zhang, Danqi Zeng, Jun Duan

**Affiliations:** 1 Key Laboratory of South China Agricultural Plant Molecular Analysis and Genetic Improvement & Guangdong Provincial Key Laboratory of Applied Botany, South China Botanical Garden, Chinese Academy of Sciences, Guangzhou 510650, China; zhaoconghui@scbg.ac.cn (C.Z.); zhenming311@scbg.ac.cn (Z.Y.); hechunmei2012@scbg.ac.cn (C.H.); wanghaobin17@scbg.ac.cn (H.W.); cans2013@163.com (C.S.); zhangmingze@scbg.ac.cn (M.Z.); zengdanqi20@scbg.ac.cn (D.Z.); 2 College of Life Sciences, University of Chinese Academy of Sciences, No. 19A Yuquan Road, Shijingshan District, Beijing 100049, China; 3 Center of Economic Botany, Core Botanical Gardens, Chinese Academy of Sciences, Guangzhou 510650, China; 4 Independent Researcher, P.O. Box 7, Miki-Cho Post Office, Ikenobe 3011-2, Kagawa-ken 761-0799, Japan; jaimetex@yahoo.com

**Keywords:** floral volatiles, geraniol, MEP pathway, orchids, terpene synthase

## Abstract

Floral scent is a key ornamental trait that determines the quality and commercial value of orchids. Geraniol, an important volatile monoterpene in orchids that attracts pollinators, is also involved in responses to stresses but the geraniol synthase (GES) responsible for its synthesis in the medicinal orchid *Dendrobium officinale* has not yet been identified. In this study, three potential geraniol synthases were mined from the *D. officinale* genome. DoGES1, which was localized in chloroplasts, was characterized as a geraniol synthase. *DoGES1* was highly expressed in flowers, especially in petals. *DoGES1* transcript levels were high in the budding stage of *D. officinale* flowers at 11:00 a.m. DoGES1 catalyzed geraniol in vitro, and transient expression of *DoGES1* in *Nicotiana benthamiana* leaves resulted in the accumulation of geraniol in vivo. These findings on DoGES1 advance our understanding of geraniol biosynthesis in orchids, and lay the basis for genetic modification of floral scent in *D. officinale* or in other ornamental orchids.

## 1. Introduction

Plants emit an astonishing number of volatile metabolites during growth and development, and these have various roles, some with biological effects, that are considered beneficial to plants and humans [1]. For ornamental plants, floral volatiles have a dual function, to attract pollinators, and in defense against pests, herbivores, and pathogens [2,3,4]. Orchids, economically important floricultural crops, possess an abundance of floral volatile terpenes. Among them, monoterpenes, especially geraniol, linalool, and their oxygenated derivatives, are predominant components of floral scents [4,5]. Geraniol is an acyclic monoterpene alcohol released from several ornamental plants, such as citronella, geranium, herbs, roses, and orchids (*Phalaenopsis bellina* and *Dendrobium officinale*) [5,6,7,8], and is extensively used in fragrance and cosmetics industries because of its pleasant rose-like scent.

Geraniol is synthesized from geranyl pyrophosphate (GPP), the universal five-carbon precursor for the biosynthesis of all monoterpenes, and is catalyzed by a terpene synthase (TPS), which has been designated as geraniol synthase (GES, EC 3.1.7.11) [6,9]. GPP, as an immediate precursor of monoterpenes, is proceeded by a condensation reaction of two C5-isoprene building units, namely isopentenyl diphosphate (IPP) and dimethylallyl diphosphate (DMAPP) [9]. Recent studies have thoroughly characterized two well-established pathways, the cytosolic mevalonic acid (MVA) pathway and the plastidic methylerythritol phosphate (MEP) pathway, that generate IPP and DMAPP, [7,8,9]. Several enzymes, including 1-deoxy-d-xylulose 5-phosphate synthase (DXS), 1-deoxy-d-xylulose 5-phosphate reductoisomerase (DXR), 4-hydroxy-3-methylbut-2-en-1-yl diphosphate synthase (HDS), and GPP synthase (GPPS), contribute to GPP biosynthesis [10], providing the GPP substrate for GES to generate geraniol. Taken together, GES is a mono-TPS that specifically catalyzes the formation of geraniol from GPP in the MEP pathway.

**Figure 1:**
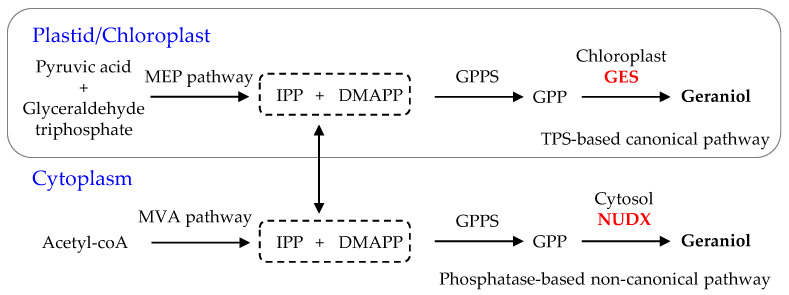
The pathway of the *GES*/*NUDX* genes responsible for the formation of geraniol in planta [9,10,11]. C5 precursors DMAPP and IPP are generated by the cytosol mevalonic acid (MVA) and the plastid methylerythritol phosphate (MEP) pathways. DMAPP, dimethylallyl pyrophosphate; GES, geraniol synthase; GPP, geranyl pyrophosphate; GPPS, GPP synthase; IPP, isopentenyl pyrophosphate; NUDX, nudix hydrolase; TPS, terpene synthase.

In plants, two kinds of enzymatic reactions can produce geraniol from GPP, either a TPS-based canonical pathway, which is catalyzed by GES in chloroplasts/plastids [6], or a phosphatase-based non-canonical pathway, which is catalyzed by nudix hydrolase (NUDX) in the cytoplasm (Figure 1) [9,11]. Thus far, the *GES* gene has already been identified and functionally characterized in multiple horticultural plants, including *CitTPS16* in *Citrus sinensis* [12], *LoTPS3* in *Lathyrus odoratus* [13], *GES* in *Ocimum basilicum* [6], and *PbGDPS* in *P. bellina* [14], all of which can produce geraniol from GPP in vitro. However, no TPS with GES activity has been identified in *Rosa rugosa* to date. Only one *NUDX* gene, *RhNUDX1*, converts GPP into geranyl monophosphate (GP), which is then hydrolyzed to geraniol by a petal-derived phosphatase [11]. In orchids, *PbGDPS*, which encodes GPP synthase, may play a key role in regulating the biosynthesis of monoterpenes (geraniol and linalool) in *P. bellina* [14]. In addition, the transcript levels of two TPS genes (*PbTPS5* and *PbTPS10*) are consistent with the production of geraniol and linalool in *P. bellina* [15], although their functionality has not yet been verified. Although geraniol is an important floral volatile compound in *D. officinale*, a medicinal orchid [7], the *GES* gene responsible for geraniol biosynthesis in *D. officinale* has not yet been characterized.

Herein, using the *D. officinale* genome database [16,17], and according to phylogenetic analysis and sequence homology, three *GES* genes (named *DoGES1–3*), with putative roles in the production of geraniol, were screened. The transcriptional regulatory functions of *DoGES1*, a member of the TPS family, in response to the accumulation of geraniol in *D. officinale* was investigated in different plant tissues (roots, stems, leaves, and flowers), harvest times (8:00, 11:00, 14:00, and 17:00), flower organs (petals, sepals, and gynostemium), and flowering periods (budding, semi-open flowers, fully open flowers). An in vitro assay of recombinant protein in *Escherichia coli* BL21 star (DE3) as well as in vivo transient overexpression in *Nicotiana benthamiana* indicated that *DoGES1* was responsible for geraniol biosynthesis, advancing our understanding of geraniol biosynthesis in *D. officinale*.

## 2. Results

### 2.1. Identification of Candidate GES Genes from the D. officinale Genome

**Figure 2:**
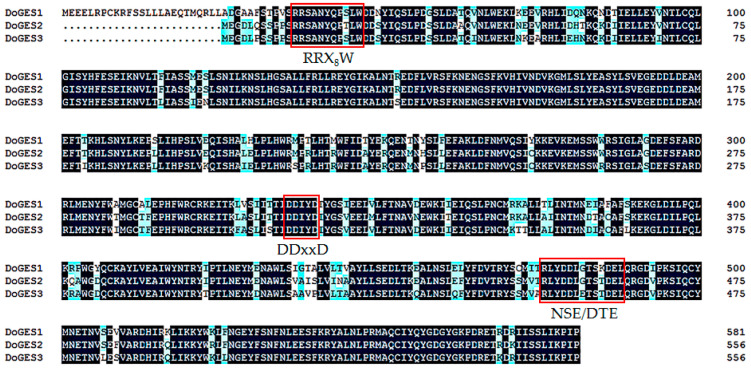
Comparison of deduced amino acid sequences of DoGES proteins in *Dendrobium officinale*. The Asp-rich domain DD_XX_D, the RRX_8_W motif, and the NSE/DTE motif, which are highly conserved in plant TPS proteins and required for TPS activity, are indicated. Completely conserved sequences are shaded in black, identical sequences in dark grey, and similar sequences in light grey.

From *D. officinale* genomic annotation data, three candidate GES sequences with best matches to known GES proteins [6,12,13,15] were retrieved by BLASTN, and named DoGES1, DoGES2, and DoGES3 (Table S1). Multiple sequence alignment demonstrated that three DoGES proteins had highly conserved aspartate-rich motifs (DDxxD) and NSE/DTE motifs at the *C*-terminal, and an RRX_8_W domain at the *N*-terminal (Figure 2), suggesting that DoGES1-3 were all TPSs. Among them, DDxxD and NSE/DTE were essential for the cofactor Mg^2+^ or Mn^2+^ to catalyze the synthesis of monoterpenes [18,19], and the RRX_8_W domain was also involved in the cyclization of monoterpene synthase [20].

### 2.2. Phylogenetic Analysis of DoGES Proteins in the D. officinale Genome

**Figure 3:**
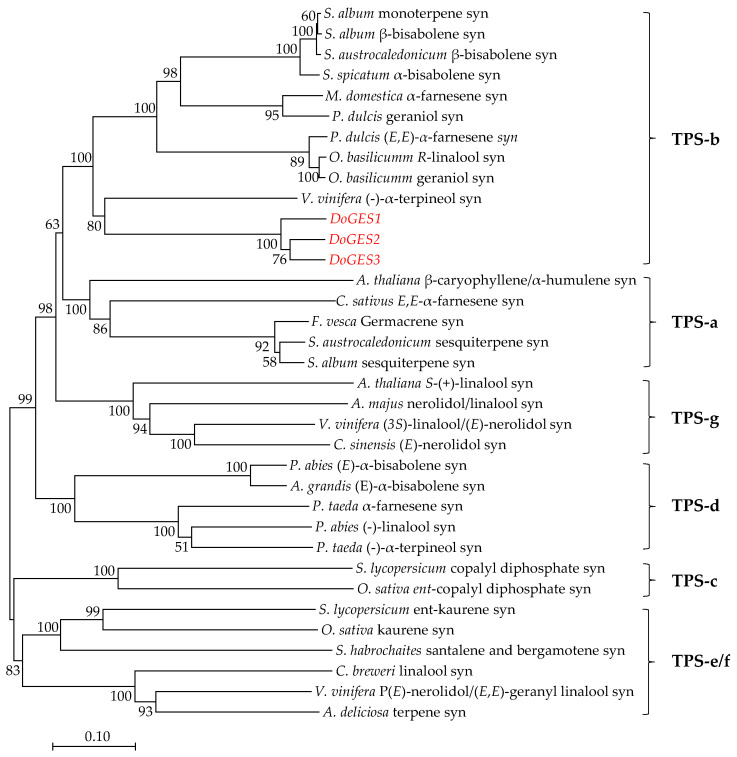
Phylogenetic positioning of GES proteins within representative samples of known plant TPS proteins. The neighbor-joining tree was generated using MEGA 7.0 software after the alignment of full-length DoGES proteins in *D. officinale* with other plant TPS proteins. The seven subfamilies TPS-a-g are delimited based on the taxonomic distribution of the TPS families [19]. All sequences that were used can be retrieved from Supplementary Table S2. syn, synthase.

To investigate the evolutionary relationship of DoGES proteins with other reported GES proteins, a phylogenetic tree was generated by the neighbor-joining method (Figure 3; Table S2). All three DoGES proteins clustered in the TPS-b subfamily, which is specific to angiosperms and is responsible for encoding monoterpene synthases [21].

Based on the transcription levels in different tissues (roots, stems, leaves, and flowers), *DoGES1* exhibited high expression in flowers, while *DoGES2* and *DoGES3* were mainly expressed in roots and leaves, respectively (Figure S1). Consequently, DoGES1 was selected for our candidate study gene related to floral scent formation.

### 2.3. Molecular Cloning and Analysis of DoGES1 from D. officinale Flowers

RNA isolated from *D. officinale* flowers during the blossoming period were used as template and amplified via nested PCR. Full-length cDNA sequences of *DoGES1* have a 1749-bp long open reading frame (ORF) that encodes 582 amino acids with a theoretical isoelectric point of 5.34 and a molecular weight of 67.99 kDa (Figure S2). The DoGES1 sequence was submitted to GenBank Data Libraries under accession number MT875214.

The DoGES1 secondary structure, which was determined using the SOPMA program (http://npsa-pbil.ibcp.fr/), shows that it harbors 69.24% α-helixes, 23.37% random coils, 3.78% β-turns, and 3.61% extended strands. The Chlorop 1.1 tool predicted that DoGES1 contains a 34 amino acid long *N*-terminal chloroplast transit peptide. To determine the subcellular localization of DoGES1, three subcellular localization tools (AtSubP [22], Plant-mPLoc [23], and pLoc-mPlant [24]) were used. All of them demonstrated that DoGES1 was located in chloroplasts, and was thus likely a mono-TPS in the MEP-pathway, but not in the cytosolic MVA pathway.

### 2.4. Subcellular Localization of DoGES1

**Figure 4:**
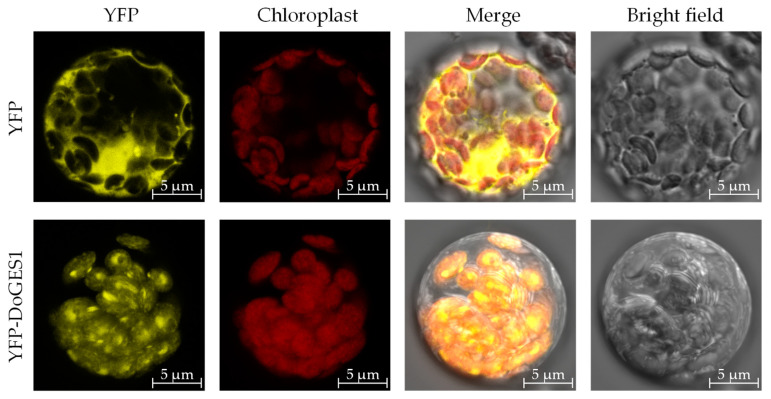
Subcellular localization of DoGES1 in *Dendrobium officinale*. Yellow fluorescence indicates the DoGES1-YFP fusion protein signal. Red fluorescence is chloroplast autofluorescence. The merged images indicate a combination of chloroplast autofluorescence and YFP fluorescence.

To confirm the intracellular localization of DoGES1, pSAT6-EYFP-DoGES1 was transformed to the mesophyll protoplasts of 4-week-old *Arabidopsis thaliana* leaves. Yellow fluorescent signals were visualized by confocal laser scanning microscopy. The images indicate that DoGES1 was located in chloroplasts (Figure 4), similar to *LiTPS2*, which encodes a mono-TPS in lily (*Lilium longiflorum* ‘Siberia’) [25], indicating that DoGES1 may be responsible for monoterpene synthesis.

### 2.5. Functional Characterization of Enzyme Encoded by DoGES1 in Escherichia coli

**Figure 5:**
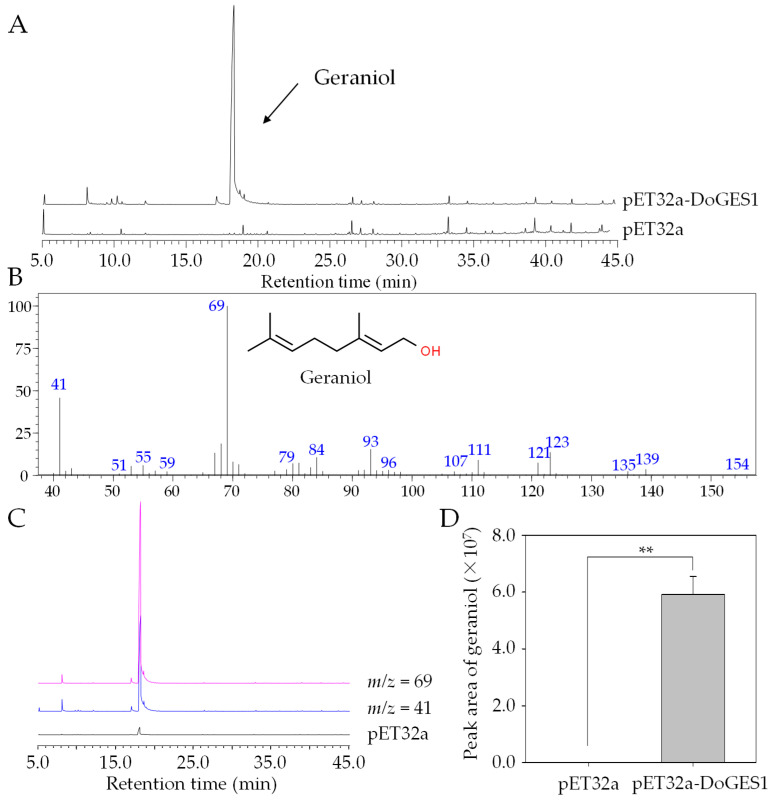
In vitro enzymatic assays of recombinant DoGES1 using GPP as the substrate. (**A**) Total ion chromatogram of the products formed by incubating extracts of empty vector pET32a and pET32a-DoGES1 with GPP. (**B**) Mass spectrum of products generated by the pET32a-DoGES1 enzyme. It is almost identical to the mass spectrum of geraniol, the standard. (**C**,**D**) Gas chromatograms of products yielded by DoGES1 using GPP as substrate. *m*/*z*, mass-to-charge ratio. In (**D**), different letters above error bars (standard deviation) (*n* = 3) indicate significant differences (** indicates *p*  <  0.01, Student’s *t*-test) between pET32a and pET32a-DoGES1.

To investigate whether DoGES1 encodes an enzyme that can produce monoterpenes, the full-length ORF sequence of DoGES1 was subcloned into prokaryotic expression vector pET-32a, expressed in *E. coli* BL21 (DE3), and induced by 1 mM isopropyl-β-d-thiogalactopyranoside (IPTG). Recombinant pET-32a-DoGES1 proteins were purified using affinity chromatography on a Ni-NTA agarose column, and were identified and isolated with a single band matching the expected size of DoGES1 in an SDS-PAGE gel (Figure S3). After incubation with GPP as substrate for the synthesis of monoterpenes, recombinant DoGES1 proteins successfully yielded geraniol with two characteristic fragment *m*/*z* 69 and *m*/*z* 41 in mass spectra produced by GC–MS (Figure 5), which showed the same mass spectral features of the authentic standard, geraniol. In addition, geraniol was not detected among the protein extracts from an empty vector cell mixture (Figure 5). These results suggest that DoGES1 from *D. officinale* had the capacity to specifically catalyze the formation of geraniol. Consequently, DoGES1 was classified as a geraniol synthase.

### 2.6. Ectopic Expression of DoGES1 in N. benthamiana

**Figure 6:**
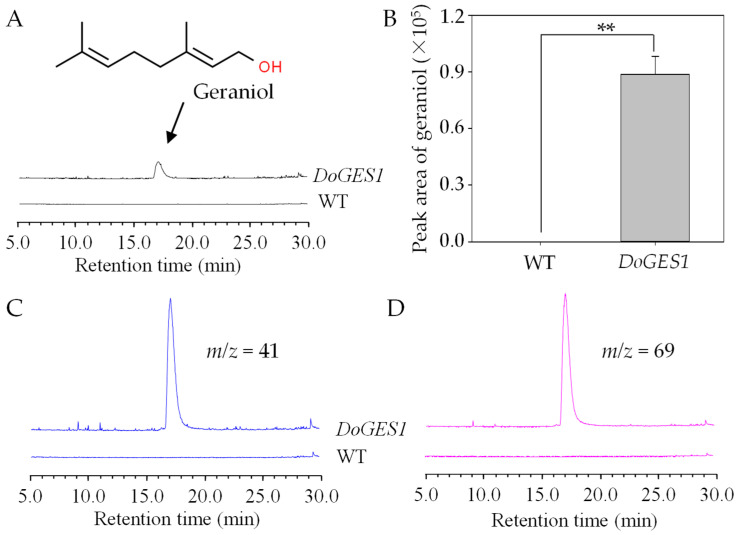
Ectopic expression of *DoGES1* in *Nicotiana benthamiana* leaves. (**A**,**B**) GC-MS analysis of monoterpenes from *N. benthamiana* leaves overexpressing *DoGES1*. The *N. benthamiana* leaves transformed with pCAMBIA3300 served as the control group (WT). (**C**,**D**) Mass spectrum of products generated in *N. benthamiana* leaves overexpressing *DoGES1*. In (**B**), error bars (standard deviation) (*n* = 3) indicate significant differences between WT and treatments (** indicates *p*  <  0.01, Student’s *t*-test). *m*/*z*, mass-to-charge ratio. WT, wild type.

We ectopically expressed *DoGES1* under the control of the cauliflower mosaic virus (CaMV) 35S promoter in *N. benthamiana* leaves. Positive transgenic leaves were screened by PCR for the presence of the *DoGES1* gene. *DoGES1* was not detected in 6-week-old *N. benthamiana* leaves transformed with the empty vector pCAMBIA3300, while the positively transformed *N. benthamiana* leaves were selected for subsequent analysis as a result of the high transcription levels of *DoGES1*. As expected, a large amount of geraniol was produced in *N. benthamiana* leaves overexpressing *DoGES1* 3 days after treatment, whereas geraniol was not observed in the control group (Figure 6). Therefore, *DoGES1* seemed to be a single-product enzyme that contributed to the biosynthesis of geraniol.

### 2.7. Temporal-Spatial Expression Patterns Analysis of DoGES1 in D. officinale

**Figure 7:**
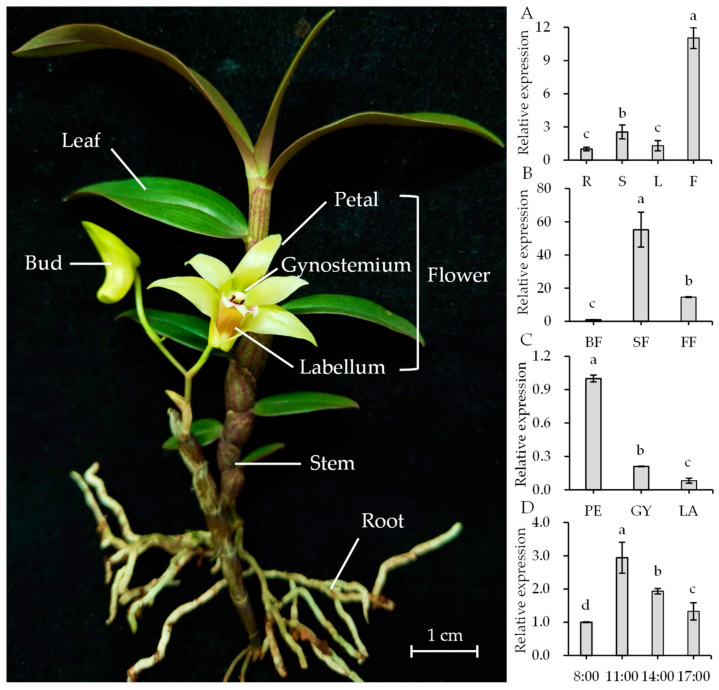
Expression levels of *DoGES1* in different *Dendrobium officinale* tissues. (**A**) Transcript levels of *DoGES1* in roots, stems, leaves and flowers. (**B**) Transcript levels of *DoGES1* in three developmental stages of *D. officinale* flowers. (**C**) Transcript levels of *DoGES1* in three *D. officinale* flower organs. (**D**) Transcript levels of *DoGES1* among the harvest time points. R, roots; S, stems; L, leaves; F, flowers; BF, budding flowers; SF, semi-open flowers; FF, fully open flowers; PE, petal; GY, gynostemium; LA, labellum. Three developmental stages of *D. officinale* flowers include budding, semi-open flower, and fully open flower are shown in Figure S6. Four time points (8:00, 11:00, 14:00, and 17:00) indicate the time of day when petals were sampled. Different letters above error bars (standard deviation) (*n* = 10) indicate significant differences among different treatments (*p* < 0.05, Duncan’s multiple range test).

To clarify the expression patterns of *DoGES1* in *D. officinale*, different tissues (roots, stems, leaves, and flowers), developmental stages of flowers (budding, semi-open flowers, fully open flowers), flower organs (petal, gynostemium, and labellum), and flower harvest times (8:00, 11:00, 14:00, and 17:00) were measured using real-time quantitative PCR (RT-qRCR). The results indicate that *DoGES1* exhibited the highest transcription levels in flowers, followed by stems, while the roots and the leaves displayed a relatively low level (Figure 7A). As flowers developed, the expression levels of *DoGES1* mRNA also varied. *DoGES1* was slightly expressed in the bud stage, expression increased enormously in semi-open flowers where it peaked, and dropped notably in the fully open flowers (Figure 7B). Among different flower organs, *DoGES1* expression was 4.77- and 12.29-fold higher in petals than in the gynostemium or labellum, respectively (Figure 7C). A comparison of *DoGES1* expression at specific times of the day showed that *DoGES1* showed higher expression at 11:00 than at 8:00, 14:00, and 17:00 (Figure 7D). Furthermore, geraniol content fluctuated among the harvest time points (from 8:00 to 17:00), with a trend of first increasing and then decreasing; the highest amount was observed at 14:00 (Figure S4). Thus, *DoGES1* was upregulated at first, then downregulated and possessed the highest value at 11:00, corresponding to the increase and then decrease in geraniol content at the same harvest time points (from 8:00 to 17:00). These results suggest that floral monoterpenes, such as geraniol encoded by *DoGES1*, might originate from petals and are emitted at 14:00, with a considerably high release during the budding stage in *D. officinale* flowers.

### 2.8. Activation of DoGES1 Gene Expression in Response to Methyl Jasmonate

**Figure 8:**
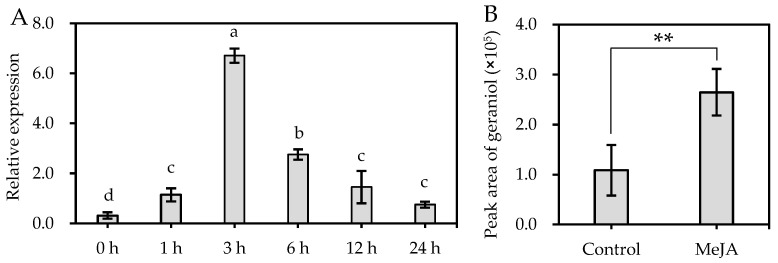
Transcript accumulation of the *DoGES1* gene (**A**), and the accumulation of geraniol (**B**) in response to methyl jasmonate (MeJA). The semi-open flowers of *Dendrobium officinale* were sprayed with 100 μM MeJA for 24 h. In (**A**), different letters above error bars (standard deviation) (*n* = 10) indicate significant differences under MeJA treatment for 24 h (*p* < 0.05, Duncan’s multiple range test). In (**B**), error bars (standard deviation) (*n* = 10) indicate significant differences between the control and MeJA treatment (** indicates *p*  <  0.01, Student’s *t*-test).

Methyl jasmonate (MeJA), which is involved in responses to various stresses, regulates terpene metabolism [26]. To explore the response of the *DoGES1* gene after the application of MeJA, *DoGES1* transcript levels were quantified by qRT-PCR. Compared to the non-treated control, *DoGES1* was significantly upregulated between 2.41- and 21.49-fold, with the highest expression at 3 h after MeJA treatment (Figure 8A). Furthermore, a significant increase (*p* < 0.05) in geraniol was induced by MeJA, increasing by 243.47% (Figure 8B). This finding suggests the involvement of *DoGES1* in the MeJA-dependent biosynthesis of geraniol.

## 3. Discussion

Geraniol, an important acyclic monoterpene with a distinctive rose-like scent, is widely used in the flavor and fragrance industries [27]. In plants, there are two biosynthetic pathways of geraniol (Figure 1). One depends on GES, and is a rate-limiting enzyme located in chloroplasts or plastids. The universal five-carbon precursors IPP and DMAPP produce GPP by plastidic GPPS in the MEP pathway, then GPP as substrate is catalyzed by mono-TPS via a common ionization-dependent reaction [28,29]. The *GES* gene, which was first cloned and functionally identified in sweet basil (*O. basilicum*), was shown to have the function of geraniol catalysis [6]. Subsequently, *GES* has been fairly extensively studied in many plant species, such as *C. sinensis* [12], *L. odoratus* [13], *Gardenia jasminoides* [30], *N. tabacum* [31], and *P. bellina* [15]. The second pathway for the production of geraniol is a phosphatase-based non-canonical pathway for monoterpene biosynthesis that involves NUDX [9,11]. After comparing the aroma components and the different gene expression profiles in two rose (*R. hybrida*) varieties, an important cytosolic enzyme, RhNUDX1 [11], was identified. When RhNUDX1 was incubated with GPP, this recombinant protein showed diphosphohydrolase activity and overexpression of *RhNUDX1* resulted in the accumulation of geraniol in *N. benthamiana*. Moreover, *RhNUDX1*-RNAi rose lines exhibited a relatively lower level of geraniol. Interestingly, *A. thaliana* NUDX1 efficiently hydrolyzed GPP to GP, but was not responsible for the production of geraniol while GES oversaw geraniol production [32]. Therefore, the proteins in the NUDX family might all be able to bind and act upon hydrolysis of GPP to GP, but only rose RhNUDX1 can promote the generation of geraniol [11], which is attributed to differential GES/NUDX enzyme localization [9,32,33]. Mono-TPS, located in chloroplasts/plastids, uses GPP as substrate, where GPP is also generated. In this study, a 34-aa chloroplast transit peptide was found in the *N*-terminal of DoGES1, so DoGES1 was targeted to the chloroplast (Figure 4). Therefore, chloroplast DoGES1 may function as the chloroplast/plastid-localized GES, but not as the cytoplasmic NUDX, resulting in the formation of geraniol via the canonical pathway. Similarly, two GES proteins were also located in plastids. Transient expression of *Valeriana officinalis VoGES* and *Lippia dulcis LdGES* in *N. benthamiana* leaves showed that GFP signal was located in the plastid [34], and HcTPS7 from *Hedychium coronarium* contained an 80 aa peptide in the *N*-terminal, targeting the protein to plastids [35].

The TPS family is typically divided into seven subfamilies, namely TPS-a, -b, -c, -d, -e/f, -g, and -h based on amino acid sequences and phylogeny [20]. TPS-a mainly codes for sesquiterpene synthase or diterpene synthase in monocotyledonous and dicotyledonous plants, respectively [21]. TPS-b and -g are both primarily involved in monoterpene synthesis, and TPS-b harbors an *N*-terminal RRX_8_W motif that is a key metal binding domain for divalent metal ions, while TPS-g lacks the RRX_8_W motif [29]. In the present study, an important TPS gene *DoGES1*, encoding geraniol synthase, was isolated from *D. officinale* flowers. It encodes 582 amino acids containing DDxxD and RRX_8_W motifs (Figure 2), and was clustered into the TPS-b subfamily (Figure 3), sharing 80% homology with mono-TPS of *L. longiflorum* ‘Siberia’ LiTPS2 [25]. DoGES1 recombinant protein accepted GPP as a substrate and singly generated geraniol in *E. coli* (Figure 5), which is associated with *O. basilicum* GES that utilizes GPP to uniquely generate geraniol in vitro [6]. Furthermore, *DoGES1* was ectopically expressed in *N. benthamiana* leaves, resulting in significantly increased geraniol content in transgenic leaves, compared to the control group (Figure 6). Although some TPS proteins have been shown to catalyze the formation of multiple products [25,30,31], GES is highly specific and produces only geraniol, similar to linalool synthase, which makes a single acyclic monoterpene alcohol, linalool [6,7,19,20]. Therefore, DoGES1 mainly acts as a geraniol synthase in the synthesis of floral volatiles.

In higher plants, the emission of volatile terpenes is often temporal and spatially specific [3,4,36]. For example, *PbGDPS*, which participates in the generation of geraniol and linalool in *P. bellina*, was specifically expressed in flowers and highly expressed on the fifth day after flower initiation [4,5,14]. The released of a high content of linalool in *Osmanthus fragrans* ‘Dangui’ petals is caused by the overexpression of mono-TPS (*LIS1* encoding linalool synthase) [37]. Ten species in *Maxillariinae* (Orchidaceae), such as *Maxillaria picta*, *M. cerifera*, and *M. marginata*, release volatile monoterpenes, mostly from the sepals and at the start of flowering [38]. In *D. officinale*, *DoGES1* was highly expressed in flowers, especially in petals, but had lower expression levels in leaves, stems, and roots (Figure 7). Generally, biosynthesis and emission of volatile compounds are developmentally regulated, usually enriching expression in an initial stage of development such as young leaves, unfertilized flowers, and unripe fruits [9,33]. With the continuous development of flowers, *DoGES1* substantially increased from the budding stage and peaked at the semi-open flower stage, decreasing at the fully open flower stage (Figure 6). This implies that GPP also accumulated extensively in semi-open flowers and generated an abundant amount of geraniol that was released in flowers, then, in fully open flowers, geraniol was heavily reduced, attributed to a reduction in the expression of *DoGES1*.

Environmental factors such as light or temperature normally influence the emission of volatile aroma scent [2]. The emission of *P. bellina* flower scent was regulated by light and the circadian clock [15]. In constant light (500 μmol·m^−2^ s^−1^), *P. violacea* emitted mostly monoterpenes whose levels decreased in constant darkness. PbNAC1, which regulates the synthesis of monoterpenes, interacts with long hypocotyl 5 (HY5), which is a positive transcription factor involved in the responsiveness of plants to light [5,39]. *DoGES1* demonstrated fluctuations throughout the day, increasing at 8:00, then decreasing from 11:00 to 17:00, peaking at 11:00 (Figure 7), corresponding to the increase and then decrease in geraniol content at the same harvest time points, with the highest value observed at 14:00, suggesting that the emission of monoterpenes may have a diurnal rhythm in *D. officinale* petals. In addition, the MeJA-induced biosynthesis of terpenes (for example, geraniol) was observed (Figure 8) in *D. officinale* semi-open flowers, mainly due to the upregulation of *DoGES1* expression. The same finding that MeJA treatment resulted in the enhanced expression of 24/36 *CsTPS* genes and an increase in the amount of monoterpene volatiles such as linalool, geraniol, and their derivatives, was reported in *Camellia sinensis* leaves [40]. The CGTCA-motif and three MYC motifs in the *DoGES1* promoter (Figure S5) can interact with the MYC2 transcription factor of the JA signaling pathway [41]. Thus, *cis*-elements (CGTCA-motif and MYC) in the MeJA-induced *DoGES1* gene may be able to activate the JA signaling pathway, thereby regulating the enhancement of monoterpene (geraniol), although this possibility needs to be further explored.

## 4. Materials and Methods

### 4.1. Plant Materials and MeJA Treatment

*D. officinale* ‘Zhongke 5′ plants were cultivated in a greenhouse under controlled environment conditions as mentioned previously [7] in the South China Botanical Garden, Chinese Academy of Sciences (Guangzhou, China). The roots, stems, leaves, and flowers (including petal, gynostemium, and labellum) from 2-year-old adult *D. officinale* plants (Figure 7), as well as three different flowering stages (budding, semi-open flowers, and fully open flowers, Figure S6), were sampled and stored at −80 °C. Additionally, the petals of semi-open flowers at different harvest times (8:00, 11:00, 14:00, and 17:00) were sampled and stored at −80 °C. Four-week-old *A. thaliana* Col-0 for subcellular localization and *N. benthamiana* plants for ectopic expression experiments were both grown in a growth room at 22 °C with a 16 h photoperiod. For the MeJA treatment, *D. officinale* flowers during the semi-open flower stage were sprayed with 100 μM MeJA for 0, 1, 3, 6, 12, and 24 h; 0 h was used as the control group. All samples were harvested, frozen in nitrogen liquid, and stored at −80 °C.

### 4.2. Molecular Cloning of the DoGES1 Gene and Bioinformatics Analysis

Based on the annotated *GES* gene sequence from the *D. officinale* genome database, one gene (*DoGES1*) was screened and cloned. Total RNA from 0.1 g of *D. officinale* flowers for gene cloning were isolated by the Quick RNA Isolation Kit (Huayueyang, Beijing, China) following the manufacturer’s protocol. The first strand cDNA of *DoGES1* was synthesized using the Reverse Transcription System (Promega Co., Madison, WI, USA) according to the manufacturer’s instruction. Full-length ORF sequences of *DoGES1* were cloned using the KOD-plus Mutagenesis Kit (Toyobo, Osaka, Japan). The PCR program was as follows: 98 °C for 2 min, 35 cycles of 98 °C for 10 s, 60 °C for 20 s and 72 °C for 30 s, then constant 72 °C for 5 min. PCR products were recovered using the HiPure Gel Pure Micro Kit (Magen, Guangzhou, China). Specific primers are listed in Table S3.

The DoGES1 amino acid sequences were submitted to Clustal X 2.0 to conduct multiple sequence alignment [42]. A phylogenetic tree was constructed by MEGA 7.0 software [43] based on the neighbor-joining computational method [44]. The secondary structure of the DoGES1 protein was determined using the SOPMA program (http://npsa-pbil.ibcp.fr/). In order to study the subcellular localization of the DoGES1 protein, AtSubP [22], Plant-mPLoc [23], and pLoc-mPlant [24], all possessing good accuracy (>70%), were used on their corresponding websites.

### 4.3. Prokaryotic Expression and Purification of DoGES1 Protein

To express the DoGES1 protein in *E. coli*, the ORF of *DoGES1* was ligated into the pET32a vector using the InFusion^®^ HD Cloning Kit (Takara, Dalian, China). The recombinant plasmid pET32a-DoGES1 was transformed into *E. coli* BL21 (DE3) competent cells (TsingKe Bio., Guangzhou, China). The identified positive clones were incubated at 37 °C with shaking at 180 rpm for 8 h in Luria–Bertani liquid medium supplemented with 50 μg mL^−1^ kanamycin. Culture medium was diluted to OD_600nm_ between 0.5 and 0.6 and placed at 37 °C. Then, 1 mM isopropyl-β-d-thiogalactopyranoside (IPTG) was added to cultures, which were incubated for 8 h at 18 °C with shaking at 160 rpm. Finally, cell cultures were harvested by centrifugation at 12,000× *g* for 10 min. The precipitate was resuspended in fresh lysis buffer (10 mM imidazole, 300 mM NaCl, 50 mM NaH_2_PO_4_, pH 8.0) and lysed by sonication (60 Hz, repeated cycles of 5 s sonication and 5 s suspension) for 40 min. The clear lysate was collected by centrifugation at 10,000× *g* for 15 min.

Protein was purified with Ni-NTA Agarose (Qiagen, Hilden, Germany), eluted with elution buffer (250 mM imidazole, 300 mM NaCl, 50 mM NaH_2_PO_4_, pH 8.0), then desalinated in PD-10 desalting columns (GE Healthcare, Chicago, IL, USA) as previously described [7]. Purified DoGES1 protein was examined by sodium dodecyl sulfate polyacrylamide gel electrophoresis (SDS-PAGE).

### 4.4. In Vitro Enzyme Assay of DoGES1

The reaction mixture consisted of 100 µg of DoGES1 protein and 1 mL of MOPSO buffer (10 mM, pH 7.0 containing 5 mM dithiothreitol, 10 mM MgCl_2_, and 10 mM GPP as substrate). The mixtures were overlaid with hexane in a total volume of 200 µL and incubated at 30 °C for 1 h. Reaction products were extracted by mixing vigorously for 5 min to obtain the enzymatic products, and 1 µL of the dehydrated extract was collected for gas chromatography-mass spectrometry (GC-MS) analysis.

### 4.5. Transient Expression of DoGES1 in *N. benthamiana*

The complete ORF sequences of DoGES1 (excluding the termination codon) were digested with *Bam*HI (Takara) and *Sac*I (Takara), and subcloned into the plant binary expression vector pCAMBIA3300 (CAMBIA, Canberra, Australia). The DoGES1-pCAMBIA3300 recombinant plasmid was transformed into *Agrobacterium tumefaciens* strain GV3101 cells via the freeze-thaw method as previously described [45]. Six-week-old leaves of *N. benthamiana* were injected with *A. tumefaciens* GV3101 cultures, and then maintained at 22 °C in darkness for 3 d. Infected 6-week-old vegetative leaves of *N. benthamiana* were collected and stored at −80 °C.

### 4.6. Quantification of Volatile Monoterpenes Using GC-MS

Volatile monoterpenes were analyzed by GC-MS equipped with a 30-m Supelcowax-10 column (0.25 mm diameter × 0.25 µm film thickness). About 500 mg of infected tobacco leaves or *D. officinale* flowers were blended with 3 mL dichloromethane containing 5 nmol ethyl decanoate (Sigma-Aldrich, St. Louis, MO, USA; CAS number 110-38-3, 98% purity) as the internal standard, and incubated at 25 °C while shaking at 100 rpm for 8 h. The extraction was passed through anhydrous Na_2_SO_4_ to remove remaining water, and filtered through a 0.22 µm PVDF membrane filter (Anpel Laboratory Technologies Inc., Shanghai, China), then concentrated to 500 µL under a nitrogen flow, and subjected to GC-MS (QP2010 SE, Shimadzu Co., Kyoto, Japan) analysis. The reaction program was carried out following our previously published protocol [7]. Products were identified by comparing mass spectra and retention times against the NIST 2008 mass spectra library (https://chemdata.nist.gov/) and the mass spectrum of the standard, geraniol (Sigma-Aldrich; CAS number 106-24-1, 98% purity).

### 4.7. Subcellular Location of DoGES1 Protein

The ORF sequence of DoGES1 was cloned into pSAT6-EYFP-N1 at the *Nco*I site, which was driven by the CaMV 35S promoter. The recombinant DoGES1-YFP plasmid was transformed into 4-week-old *A. thaliana* protoplasts that were isolated from rosette leaves by PEG-mediated transformation as described previously [46]. After incubation at 22 °C for 14 h in darkness, YFP fluorescence signals were excited at 514 nm and with an emission wavelength of 527 nm using a Leica TCS SP8 STED 3× microscope (Wetzlar, Hesse, Germany).

### 4.8. Real-Time Quantitative PCR Analysis

To analyze *DoGES1* gene expression patterns, qRT-PCR was performed. Total RNA from three flowering periods (budding, semi-open flowers, and fully open flowers), different tissues (roots, stems, leaves, flowers, petals, gynostemium, and labellum), and the flowers from different sampling times (8:00, 11:00, 14:00, and 17:00) were isolated and reverse transcribed, as described above. The total reaction volume was 10 μL containing 0.4 μL of each primer, 1 μL of template cDNA, 5 μL of SYBR Green PCR Master Mix (Novogene, Beijing, China), and 3.2 μL of ddH_2_O. The PCR reaction was carried out with the LightCycler^®^ 480 Instrument (Roche Diagnostics, Mannheim, Germany) as described previously [7]. The relative abundance of DoEF-1α (GenBank accession no.: JF825419) was used as an internal standard and calculated using the 2^−∆∆CT^ method [47]. Specific primers are shown in Table S3.

## 5. Conclusions

In the present study, we identified the *DoGES1* gene from *D. officinale*. It contributed to the regulation and production of geraniol biosynthesis. DoGES1 was located in chloroplast, and could utilize GPP to singly produce geraniol in vitro. Separately, *N. benthamiana* leaves overexpressing *DoGES1* considerably accumulated geraniol in vivo, which was consistent with the main monoterpene geraniol in *D. officinale* flowers. Our work also demonstrated that *DoGES1* was highly expressed in the petals during the semi-open flower stage at 11:00, and was activated by exogenous MeJA treatment. These results indicate that DoGES1 could effectively control the biosynthesis of geraniol in *D. officinale*, laying the foundation for biotechnological modification of floral scent profiles in orchids.

## Supplementary Materials

The following are available online at https://www.mdpi.com/1422-0067/21/19/7005/s1. Table S1. Three candidate GES sequences from the *D. officinale* genome. Table S2. The reported TPS proteins from other plant species used in the phylogenetic analysis. Table S3. Gene-specific primers used in the experiments. Figure S1. Transcription levels of *DoGES1*, *DoGES2*, and *DoGES3* in different tissues. Figure S2. Agarose gel electrophoresis of cDNA amplification of the *DoGES1* gene. Figure S3. SDS-PAGE analysis of DoGES1 recombinant protein expressed in *Escherichia coli* BL21. Figure S4. Content of geraniol in semi-open *D. officinale* flowers at 8:00, 11:00, 14:00, and 17:00. Figure S5. Putative regulatory *cis*-elements in the *DoGES1* promoter. Figure S6. Different developmental stages of *D. officinale* ‘Zhongke 5′ flowers.

## Abbreviations

DMAPP	Dimethylallyl diphosphate
DXR	1-Deoxy-d-xylulose 5-phosphate reductoisomerase
DXS	1-Deoxy-d-xylulose 5-phosphate synthase
GC–MS	Gas chromatography–mass spectrometry
GES	Geraniol synthase
GPP	Geranyl pyrophosphate
GPPS	GPP synthase
HDS	4-Hydroxy-3-methylbut-2-en-1-yl diphosphate synthase
IPP	Isopentenyl diphosphate
IPTG	Isopropyl-β-d-thiogalactopyranoside
MeJA	Methyl jasmonate
MEP	Methylerythritol phosphate
MVA	Mevalonic acid
SDS-PAGE	Sodium dodecyl sulfate polyacrylamide gel electrophoresis
YFP	Yellow fluorescent protein
