# Supplementary material for: Functional Characterization of a Dendrobium officinale Geraniol Synthase DoGES1 Involved in Floral Scent Formation
Source: Int J Mol Sci. 2020 Sep 23;21(19):7005. doi: 10.3390/ijms21197005 (PMC7582308; doi:10.3390/ijms21197005)
Supplement: Supplementary file 1 [file ijms-21-07005-s001.pdf]

Supplementary Materials

Functional characterization of a *Dendrobium officinale* geraniol synthase DoGES1 involved in floral scent formation

Conghui Zhao[1,2,†], Zhenming Yu[1,3,†], Jaime A. Teixeira da Silva[4], Chunmei He[1], Haobin Wang[1], Can Si[1], Mingze Zhang[1], Danqi Zeng[1] and Jun Duan[1,3,*]

1 Key Laboratory of South China Agricultural Plant Molecular Analysis and Genetic Improvement & Guangdong Provincial Key Laboratory of Applied Botany, South China Botanical Garden, Chinese Academy of Sciences, Guangzhou 510650, China; zhaoconghui@scbg.ac.cn (C.Z.); zhenming311@scbg.ac.cn (Z.Y.); hechunmei2012@scbg.ac.cn (C.H.); wanghaobin17@scbg.ac.cn (H.W.); cans2013@163.com (C.S.); zhangmingze@scbg.ac.cn (M.Z.); (D.Z.)

2 University of Chinese Academy of Sciences, No. 19A Yuquan Road, Beijing 100049, China

3 Center of Economic Botany, Core Botanical Gardens, Chinese Academy of Sciences, Guangzhou 510650, China

4 Independent researcher, P. O. Box 7, Miki-cho post office, Ikenobe 3011-2, Kagawa-ken, 761-0799, Japan; jaimetex@yahoo.com

* Correspondence: duanj@scib.ac.cn; Tel.: +86-020-37252978

† These authors contributed equally to this work.

Supplementary Table legends

Table S1. Three candidate GES sequences from the *D. officinale* genome.

Table S2. The reported TPS proteins from other plant species used in the phylogenetic analysis.

Table S3. Gene-specific primers used in the experiments.

Supplementary Figure legends

Figure S1. Transcription levels of *DoGES1*, *DoGES2*, and *DoGES3* in different *D. officinale* tissues.

Figure S2. Agarose gel electrophoresis of cDNA amplification of the *DoGES1* gene.

Figure S3. SDS-PAGE analysis of DoGES1 recombinant protein expressed in *Escherichia coli* BL21.

Figure S4. Content of geraniol in semi-open *D. officinale* flowers at 8:00, 11:00, 14:00, and 17:00.

Figure S5. Putative regulatory *cis*-elements in the *DoGES1* promoter.

Figure S6. Different developmental stages of *D. officinale* 'Zhongke 5' flowers.

Table S1. Three candidate GES sequences from the *D. officinale* genome.

Name	Sequences
DoGES1	MEEELRPCKRFSSLLLAEQTMQRLLADGAAFSTPVSRRSANYQPSLWDDNYIQSL<br>PDGSLDATQVNLWEKLKEEVRHLIDQNKQNDTIELLEYVNTLCQLGISYHFESEIK<br>NVLTFIASSMESLSNILKNSLHGSALLFRLLREYGIKALNTREDFLVRSFKNENGSF<br>KVHIVNDVKGMLSLYEASYLSVEGEDDLDEAMEFTTKHLSNYLKEPSLIHPSLVEQ<br>ISHALHLPLHWRMPTLHTMWFIDTYEKQENTNYSLFEFAKLDFNMVQSIYKKEV<br>KEMSSWWRSIGLAGDEFSFARDRLMENYFWAMGCALEPHFWRCRKEITKLVSIIT<br>TIDDIYDTYGSIEELVLFTNAVDEWKIIEIQSLPNCMRKALLTLINTMNEIAFAFSKE<br>KGLDILPQLKRPWGYQCKAYLVEAIWYNTRYIPTLNEYMENAWLSIGTALVLTV<br>AYLLSEDLTKEALNSLELYFDVTRYSCMITRLYDDLGTSKDELQRGDIPKSIQCYM<br>NETNVSEVVARDHIRKLIKKYWKLFNGEYFSNFNLEESFKRYALNLPRMAQCIYQ<br>YGDGYGKPDRETRDRIISSLIKPIPL
DoGES2	MEGDLQSSPPSRRSANYQPTLWDDSYIQSLPDSSLDAAQVNLWEKLNEEVRHLID<br>HTKQKDIIELLEYVNTLCQLGISYHFESEIKNVLTFIASSMESLSNILKNSLHGSALLF<br>RLLREYGIKALNTREDFLVRSFKNENGSFKVHIVNDVKGMLSLYEASYLSVEGED<br>DLDEAMEFTTKHLSNYLKEPLLIHPSLVEQISHALELPLHWRMPRLHTRWFIDAY<br>ERQENMNHSLLEFAKLDFNMVQSICKKEVKEMSSWWRSIGLAGDEFSFARDRLM<br>ENYFWTMGCTFEPHFWRCRKEITKLASLITTIDDIYDIYGSVEELMLFTNAVNEWK<br>ITEIQSLPNCMRKALLAIINTMNDTACAFSKEKGLDILPQLKQAWGDQCKAYLVE<br>AIWYNTRYIPTLNEYMENAWLSVAISLVINAAYLLSEDLTKEALNSLEFYFDVTRY<br>SSMVTRLYDDLGTSTDELQRGDVPKSIQCYMNETNVSEFVARDHIRQLIKKYWRL<br>FNGEYFSNFNLEESFKRYALNLPRMAQCIYQYGDGYGKPDRETRDKIISSLIKPIPL
DoGES3	MEGDLPSSPPSRRSANYQPSLWDDSYIQSLPDSSLDATQINLWEKLNKEARHLIEH<br>NKQKDIIELLEYINTLCQLGISYHFESEIKNVLTLIASSIENLSNILKNSLHGSALLFRL<br>LREYGIKALNTSEDFLVRSFKNENGSFKVHIVNDVKGMLSLYEASYLSVEGEDDLD<br>EAMEFTIKHLSNYLKEPLLIHPSLVKQISHAIELPLHWRSPRLHTRWFIDAYERQEN<br>MNPSLLEFAKLDFNMVQSICKKEVKEMSSWRRSIGLASDEFSFARDRLMENYFWI<br>MGCTFEPHFWRCRKEITKFASLISTIDDIYDIYGSVEELVLFTNAVDEWKIIEIQSLP<br>NCMKTTLLALINTMNDIACAFLKEKGLDILPQLKRAWGDQCKAYLVEAIWYNT<br>RYTPTLNEYMDNAWLSAAVPLVLTAAYLLSEDLTKQALNSLQFYFDVTRYSSMV<br>ARLYDDLETSTDELQRGDVPKSIQCYMNETNVLESVARDHIRQLIKKYWKLLNGE<br>YFSNFNLEESFKRYALNLPRMAQCIYQYGDGYGKPDRETKDRIISSLIKPIPL

Table S2. The reported TPS proteins from other plant species used in the phylogenetic analysis.

Species	TPS	Accession ID in NCBI
Santalum album	Monoterpene synthase	ACF24767
S. album	β-bisabolene synthase	AIV42941
S. austrocaledonicum	β-bisabolene synthase	ADO87003
S. spicatum	*α*-bisabolol synthase	E3W206
Malus domestica	*α*-farnesene synthase	AAX19772
Prunus dulcis	Geraniol synthase	QEE82241
P. dulcis	(*E,E*)-*α*-farnesene synthase	QEE82244
Ocimum basilicum	Geraniol synthase	AY362553
O. basilicum	*R*-linalool synthase	AAV63789
Vitis vinifera	(-)-*α*-terpineol synthase	AAS79352
Arabidopsis thaliana	β-caryophyllene/*α*-humulene synthase	AAO85539
Cucumis sativus	(*E,E*)-*α*-farnesene synthase	Q66PX9
Fragaria vesca	Germacrene D synthase	LOC101314066
S. album	Sesquiterpene synthase	ACF24768
S. spicatum	Sesquiterpene synthase	E3W208
A. thaliana	*S*-(+)-linalool synthase	Q84UV0
Antirrhinum majus	nerolidol/linalool synthase 1	ABR24417
V. vinifera	(3S)-linalool/(*E*)-nerolidol synthase	ADR74212
Camellia sinensis	(*E*)-nerolidol synthase	KY033151
Pinus abies	(*E*)-*α*-bisabolene synthase	Q675L6
Abies grandis	(*E*)-*α*-bisabolene synthase	AAC24192
P. taeda	*α*-farnesene synthase	Q84KL5
P. abies	(-)-linalool synthase	Q675L2
P. taeda	(-)-*α*-terpineol synthase	Q84KL4
Solanum lycopersicum	Copalyldiphosphate synthase	BAA84918
Oryza sativa	*ent*-copalyl diphosphatesynthase1	Q6ET36
S. lycopersicum	*ent*-kaurene synthase	AEP82778
O. sativa	Kaurene synthase 1	Q0JA82
S. habrochaites	Santalene and bergamotene synthase	ACJ38409
Clarkia breweri	Linalool synthase 2	AAD19840
V. vinifera	P(*E*)-nerolidol/(*E,E*)-geranyl linalool synthase	NP001268004
Actinidia deliciosa	Terpene synthase	ACO40485

Table S3. Gene-specific primers used in the experiments

**Name**	**Sequences (5′→3′)**	**Description**
DoGES1-F	ATGGAGGAAGAACTTCGCCCG	Gene cloning
DoGES1-R	TAGAGGAATGGGCTTAATTA	
DoGES1-32a-F	GGCTGATATCGGATCCTATGGAGGAAGAACTTCGCCCG	pET32a vector construction
DoGES1-32a-R	GTGCGGCCGCAAGCTTTAGAGGAATGGGCTTAATTA	
DoGES1-3300-F	CTCGGTACCCGGGGATCCTATGGAGGAAGAACTTCGCCCG	pCAMBIA 3300 vector construction
DoGES1-3300-R	GGCCAGTGCCAAGCTTTAGAGGAATGGGCTTAATTA	
DoGES1-YFP-F	ATTTACGAACGATAGCCATGGCTATGGAGGAAGAACTTCGCCCG	YFP vector construction
DoGES1-YFP-R	AGATCTGAGTCCGGACCATGGTTAGAGGAATGGGCTTAATTA	
DoGES1-qRT-PCR-F	GCCTTTACGAGGCTTCTTATCT	qRT-PCR analysis
DoGES1-qRT-PCR-R	TTGTTCCACTAACGAGGGATG	
DoGES2-qRT-PCR-F	GCTTATGAGAGGCAAGAGAACA	qRT-PCR analysis
DoGES2-qRT-PCR-R	CCACCAACTAGACATCTCCTTAAC	
DoGES3-qRT-PCR-F	TGATGCTTATGAGAGGCAAGAG	qRT-PCR analysis
DoGES3-qRT-PCR-R	TCATCGCTAGCAAGACCAATAC	
DoEF-1α-F	GCTTGAGAAGGAGCCCAAGT	qRT-PCR analysis
DoEF-1α-R	CCAACAGCCACAGTTTGTCG	

Gene-specific primers for real-time quantitative reverse transcription PCR (qRT-PCR) were designed by the PrimerQuest online tool (http://www.idtdna.com/Primerquest/Home/Index). The *D. officinale* actin gene (DoEF-1α) was obtained from NCBI (JF825419). F, forward; R, reverse.

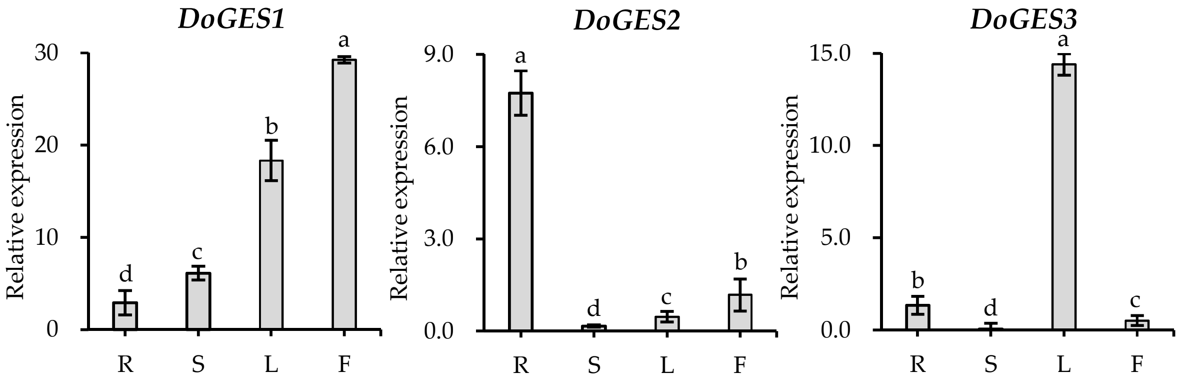

Figure S1. Transcription levels of *DoGES1*, *DoGES2*, and *DoGES3* in different *D. officinale* tissues. Different letters above error bars (standard deviation) (n = 10) indicate significant differences between treatments ($p < 0.05$, Duncan's multiple range test). R, roots; S, stems; L, leaves; F, flowers.

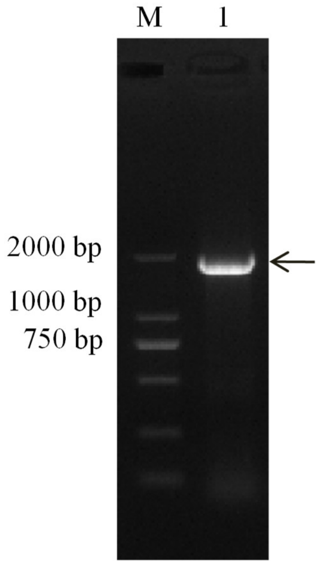

Figure S2. Agarose gel electrophoresis of cDNA amplification of the *DoGES1* gene. M, marker D2000 (M122, GenStar Inc., Beijing, China).

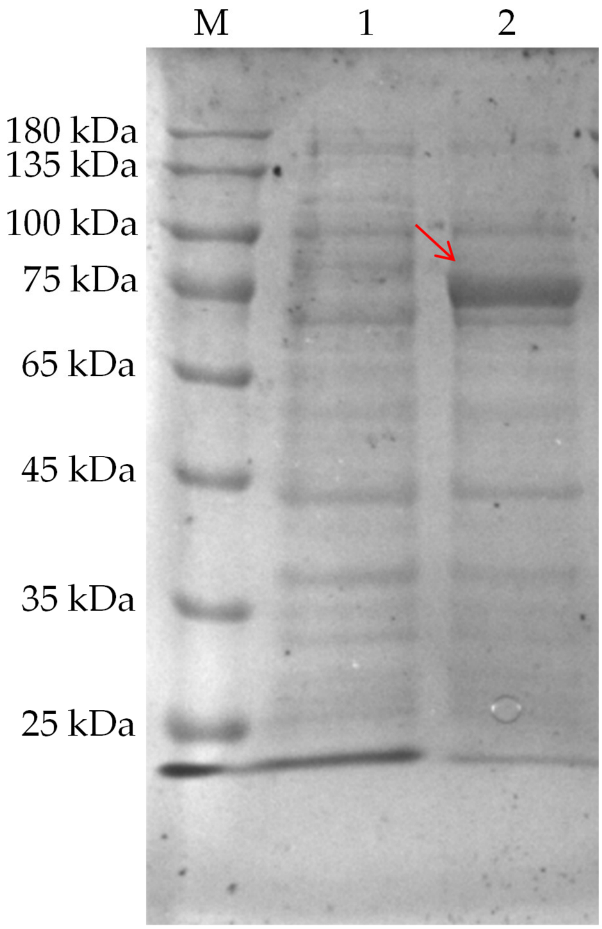

Figure S3. SDS-PAGE analysis of DoGES1 recombinant protein expressed in *Escherichia coli* BL21. Lane 1 indicates empty vector pET32a. Lane 2 indicates the elute of pET32a-DoGES1 protein. M indicates protein marker 10-180 kDa (M221, GenStar Inc., Beijing, China).

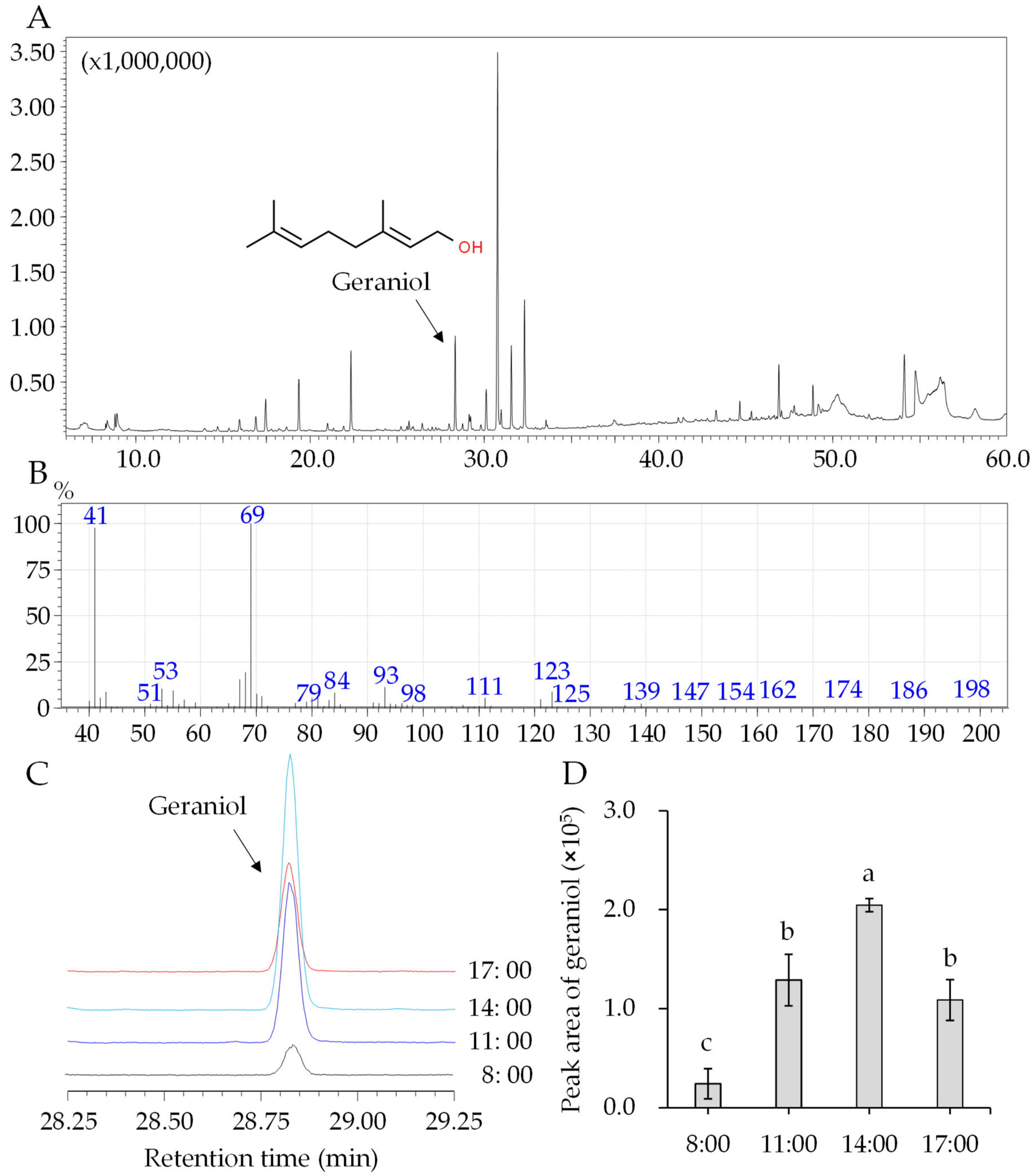

Figure S4. Content of geraniol in *D. officinale* semi-open flowers at 8:00, 11:00, 14:00, and 17:00. Each bar represents the mean (± standard error, $n = 10$) of three independent biological replicates. Different letters above the bars indicate significant differences ($p < 0.05$, Duncan's multiple range test).

TTGGAGGATTGTAGTCGTCGACCAACGGAGATTTGAAGAATCATAATTGAGCGATGATGGTTGACTATCGACGAAGGTT
AGATTTATCATAGTCGGGCAACTAAGGTTGAAATATCATATTTGGTAGTTGATGGAGGTCGTTAGTATGAAAGCTTTATAA
ATGTTTAAATTTAAATAAGATAATATAGTCTTTTGATATTTGTTAATGGAACGTGAATATTTTTAAAGACATGTTCCTTGAC
TTTTATGCGCTTTGTTAGTCATTGAATACTATTGTCAAAATAATTGATATTATAATCTCTTTAAACAATCAAATTTTAGGCAT
AATTATATTCATCCATATAGATTTATATAATAGTTTAAAAAGTGACAACTAATAATAAATATTTAATTAAATTATTTACAAA
ATTTATTTTAATATAAAATTTAATAATGAAGTCATCTAATTCACCACATATAATTGAAGAGCTAAGAGAAATAAAAAAAA
GGAGGGAGATTCAAGAGCTAAGAGAAATAAAGA**ATTCTCTT**GTATTTTCCAAGCTATCATCGTCGCTG**ACTGC**TGCCA
GCAATCGCCGCCAGCATTGTCTGACTTCTATCAATATTTAACTCAGTTATTGAATTTTAATCATCCTCTTATAGATATCAAA
TTTTTGTAATTCCTACAAGAAAACTTGG**CAATTG**TTCTTAATTCTAAATAATCCTATCTTCAGCAAATGTGAAGAAGGGA
AGATATATTATTGTATCTCATATTAATGCAAAAAAATAGGATGATGAAAGGATACCATAAAAAATTCATACAATCGGCACA
AATCTCGATATATATTTGAATTGAAAACCTTCAAAATGAAGTTTTTGATCATCCAAACTTTGAGTTTAACAAACTTATATT
CGAAGTGAAGTGTTGATGAAACGAATCTACCGATGCTACAAAATGATTCAATCCAATTCAAAATGAGCTCAAATCAAG
CTCTTAAGTTATGTTTGGAAACTTTTTAATATAAATTAATTGATCGAACAACCTCCAATCAAAGCATGCTCATATTCAACT
CGAGCAACTAACGAGCCAAATCTAACAAGCACAAGATCACTTGATTTGAACATGATTAAGACATTCAAATATTTGATAA
ACAATCCACGGTCAACAAGATCAATATGGACTGTACGATGAAATGCCTTGACTTATGGCCAAATTAACCAAAATGTTCA
TCTCAAAAAGCTTATAAATAGTGCCTATGGAGGTTCAATAGTTGAGAAAATTAAATA**ATTCTCTT**CTACTTCCTAACTGT
GTCATAGCCAACTAACACCACTAACAATTATCATCACCACCGCCAAGCTTTTTTCAACATATAGACATATTGTTAGGCTT
TTTTAATCATCTTTTTATGAACATTAAGTTTCTATAATTCCTACAAAGAAACTCCGATAGTTATTCTCAATCATAAATAATC
TCATATTTTCCAAACTATGACGAAGGGAAGATTCTTATATCTTATATTATATAATGGATTATTATAATTAGGCTAAGTCTTCT
TGGATTTTTTTTTTCAATAGACAGGATATTAAACAACAATTCTACCGACACAAATTTTTTAGCAAGTAACAGTAGTAATTG
ATAAAAATTAAAATATAAATGAAATAAAATTAAAATCACTAAATATTATTGAATTATTGAGAAATAAATAAAAGTCAATA
CAAAAAGGTTTTTTCCAAGTCTTCTTAAACTAATCAAGCACTAAAATGAAAAATTAGTCTCATTCTGACGGCCATGCAT
GTAAAATGCTTTTCACATTCACGAAATAACATATTAATCGAGTTGGGTGAAAGCAACTTTTGGAAGGTAAGTCAAATCC
CAAATTTTCTTAATAAAATAAAACAGAGCTACAAACAAGTGGGTTATAAATTATGATTGAAACAACTACGTTCAGAGCA
GAGATATTGCTTCATTAGGCCTTTTCCAGCTTTCCTTCTTCATTTCCCCTTCTGTATTCTTTAATAAGAAGA**ATG (position 0)**

Figure S5. Putative regulatory *cis*-elements in the DoGES1 promoter. The CGTCA-motif is indicated in red at the -1440 position. The MYC motif is indicated in blue at +675, -731, and -1473 positions. PlantCARE (http://bioinformatics.psb.ugent.be/webtools/plantcare/html/) was used for the promoter analysis. The initiation codon of DoGES1 gene (ATG) is set as position 0.

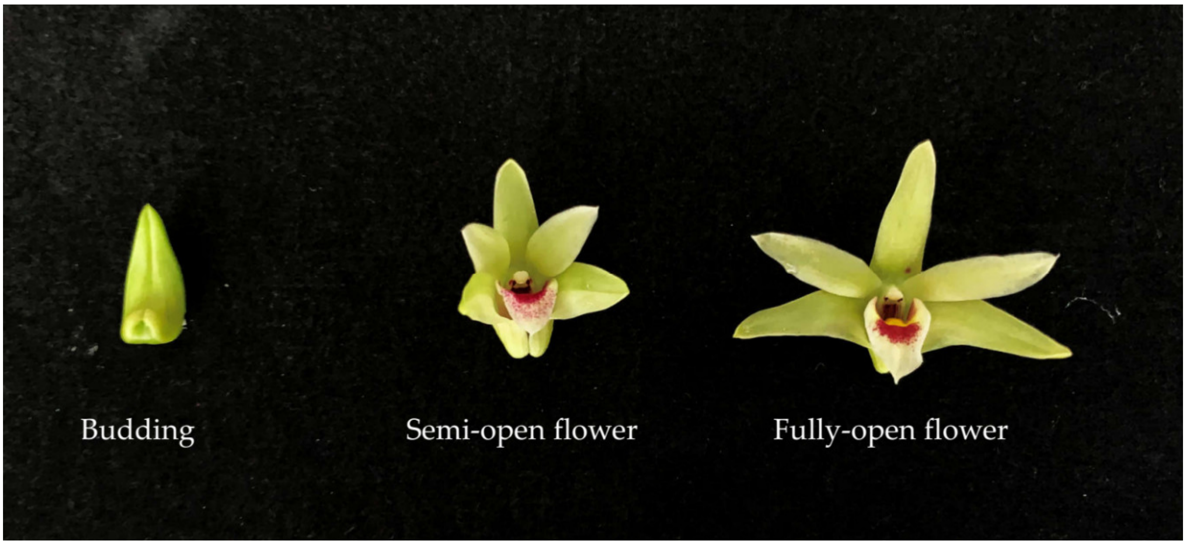

Figure S6. Different developmental stages of *D. officinale* 'Zhongke 5' flowers. Three developmental stages of *D. officinale* 'Zhongke 5' flowers, includes budding, semi-open flower, and fully-open flower.
